# Splanchnic and Systemic Hemodynamics in Cirrhotic Patients With Refractory Ascites. Effect of Peritoneovenous Shunting

**DOI:** 10.1155/1991/16710

**Published:** 1991

**Authors:** Corinne Vons, Antoine Hadengue, Samuel S. Lee, Claude Smadja, Dominique Franco, Didier Lebrec

**Affiliations:** 1 Groupe de Recherche sur la Chirurgie du Foie et de l'Hypertension Portale: Hôpital Louise Michel Evry, Hôpital Bicêtre, Le Kremlin Bicêtre, Hôpital Paul Brousse, Villejuif, and from Unité de Recherches de Physiopathologie Hépatique INSERM U 24, Hôpital Beaujon Clichy France; 2 INSERM U-24 Hôpital Beaujon Clichy 92118 France

## Abstract

The splanchnic and systemic hemodynamics of 14 patients with refractory ascites were studied and were compared to those of 15 patients with ascites responding to medical treatment. Among the 14 patients, 10 were grade B and 4 C, according to the Pugh classification. Of the 15 patients, 5 were Pugh B and 10 C. In patients with refractory ascites, free hepatic venous pressure was significantly higher and hepatic venous pressure gradient was significantly lower than in patients with responsive ascites. Hepatic and azygos blood flows were not significantly different between the two groups. Cardiac output was lower in patients with refractory ascites (p < 0.05) than in those with responsive ascites. In patients with refractory ascites, six months after peritoneovenous shunting, there was a significant reduction of wedged and free hepatic venous pressures and azygos blood flow. Cardiac output increased by 20% (p < 0.02). This study shows that hemodynamic alterations in patients with refractory ascites is the consequence of increased intraabdominal pressure due to chronic ascites. Six months after peritoneovenous shunting splanchnic and systemic hemodynamics became similar to those observed in patients without ascites.


*HPB Surgery*, 1991, Vol. 3, pp. 259–269
Reprints available directly from the publisher
Photocopying permitted by license only
© 1991 Harwood Academic Publishers GmbH
Printed in the United Kingdom

### SPLANCHNIC AND SYSTEMIC HEMODYNAMICS IN CIRRHOTIC PATIENTS WITH REFRACTORY ASCITES. EFFECT OF PERITONEOVENOUS SHUNTING

CORINNE VONS, ANTOINE HADENGUE, SAMUEL S. LEE, CLAUDE SMADJA, DOMINIQUE FRANCO and DIDIER LEBREC*

*Groupe de Recherche sur la Chirurgie du Foie et de l'Hypertension Portale: Hôpital Louise Michel, Evry, Hôpital Bicêtre, Le Kremlin Bicêtre, Hôpital Paul Brousse, Villejuif, and from Unité de Recherches de Physiopathologie Hépatique, INSERM U 24, Hôpital Beaujon, Clichy, France*

*(Received 15 July 1990)*

The splanchnic and systemic hemodynamics of 14 patients with refractory ascites were studied and were compared to those of 15 patients with ascites responding to medical treatment. Among the 14 patients, 10 were grade B and 4 C, according to the Pugh classification. Of the 15 patients, 5 were Pugh B and 10 C. In patients with refractory ascites, free hepatic venous pressure was significantly higher and hepatic venous pressure gradient was significantly lower than in patients with responsive ascites. Hepatic and azygos blood flows were not significantly different between the two groups. Cardiac output was lower in patients with refractory ascites ($p < 0.05$) than in those with responsive ascites. In patients with refractory ascites, six months after peritoneovenous shunting, there was a significant reduction of wedged and free hepatic venous pressures and azygos blood flow. Cardiac output increased by 20% ($p < 0.02$). This study shows that hemodynamic alterations in patients with refractory ascites is the consequence of increased intraabdominal pressure due to chronic ascites. Six months after peritoneovenous shunting splanchnic and systemic hemodynamics became similar to those observed in patients without ascites.

KEY WORDS: Refractory ascites, cirrhosis, peritoneovenous shunt, hemodynamics

### INTRODUCTION

In patients with cirrhosis, splanchnic and systemic hemodynamics are altered[1–4]. These changes are related to both the development of portal-systemic collateral circulation[5] and the severity of liver failure[6]. The hemodynamics of patients with cirrhosis and refractory ascites has been less well documented[7]. Although the immediate consequences of peritoneovenous shunting on systemic hemodynamics have been well demonstrated[8,9], its long term influence on splanchnic and systemic hemodynamics is poorly understood[10]. The purpose of this work was to study the

*Address for correspondence: Dr. D. LEBREC, INSERM U-24, Hôpital Beaujon, 92118 Clichy, France.

splanchnic and systemic hemodynamics before and 6 months after peritoneovenous shunting in a series of 14 consecutive patients with cirrhosis and refractory ascites in order to determine first, if preoperative measurements were similar to those of patients with ascites responding to medical treatment and second, the late hemodynamic consequences of shunting.

### PATIENTS AND METHODS

### *Patients*

Twenty-nine adult patients admitted for ascites were studied. Transvenous liver biopsy showed alcoholic cirrhosis in all patients. Among these patients, 15 responded to the medical treatment and 14 had refractory ascites. Four of the former patients had histological lesions of acute alcoholic hepatitis. Laboratory values at the time of the investigation are indicated in Table 1. The severity of cirrhosis was estimated according to Pugh's classification[11]. In patients with refractory ascites 10 were grade B and 4 C. In patients with responsive ascites, 5 were grade B and 10 C ($p < 0.01$). The medical treatment included at least bed rest, suppression of alcoholic intake, salt restricted diet (< 20 mmol/day), water restriction (< 1 l/day), spironolactone (at least 600 mg/day) and/or Frusemide (at least 200 mg/day). Ascites were considered refractory when medical treatment failed to control ascites and/or induced a decrease of natremia below 125 mmol/1 and/or an increase in creatinine blood level above 100 μmol/l. In patients with refractory ascites, ascites had been present for 3 to 13 months (mean: 7.2 ± 3.2 months). In the 15 patients with ascites responding to the medical treatment, ascites had been present for 15 days to 2 months (mean: 0.9 ± 0.7 months).

Peritoneovenous shunting was performed using the LeVeen shunt as previously described[12]. The venous catheter was introduced through the right internal jugular vein. The tip of the venous tubing was placed at the junction of the superior vena cava and of the right atrium under X-ray control. The functioning of the shunt was always tested by injecting methylen blue in the peritoneal cavity at the end of the operation and checking its appearance in the cervical portion of the venous tubing. Postoperative treatment included chest physiotherapy and abdominal binding. One dose of fruosemide (40 mg) was administered in the operating room, after the insertion of the shunt and was then discontinued.

Systemic and splanchnic hemodynamic investigations were performed in all patients. In the 14 patients with refractory ascites, the hemodynamic measurements were repeated 6 months after successful peritoneovenous shunting. At the time of the first hemodynamic study, ascites were massive in all patients.

All patients gave oral informed consent in the presence of a witness to the investigations described below.

### *Hemodynamic Studies*

The hemodynamic studies were performed in patients positioned supine after an overnight fast. Under local anesthesia, a vessel dilator with a polypropylene sheath (Desilets, Vygon, Ecouen, France) was positioned in the lumen of the right jugular vein in order to introduce successively different catheters. The gradient between

**Table 1** Laboratory values in cirrhotic patients with ascites and six months after peritoneovenous shunting

| | | | *Refractory ascites* | | |
|---|---|---|---|---|---|
| | *Responsive ascites* | *p* * | *Initial evaluation* | *Six months after peritoneovenous shunting* | *p* † |
| Number of patients | 15 | | 14 | 14 | |
| Age (years) | 55 ± 10# | NS | 53 ± 10 | | |
| Serum bilirubin (μmol/L) | 51 ± 46 | <0.05 | 23 ± 8 | 17 ± 11 | NS |
| Serum albumin (g/L) | 31 ± 4 | NS | 31 ± 5 | 37 ± 3 | <0.05 |
| Prothrombin time (% of normal) | 50 ± 12 | <0.05 | 66 ± 16 | 68 ± 15 | NS |
| Serum creatinine (μmol/l) | 87 ± 31 | NS | 92 ± 13 | 89 ± 10 | NS |
| Serum sodium (mEq/L) | 133 ± 5 | NS | 133 ± 6 | 137 ± 4 | NS |
| Severity of cirrhosis (number of patients) | | | | | |
| Pugh's grade A § | 0 | | 0 | 9 | <0.01 |
| Pugh's grade B § | 5 | | 10 | 5 | |
| Pugh's grade C § | 10 | < 0.08 | 4 | 0 | |

* Statistical analysis between responsive and refractory ascites. † Statistical analysis between refractory ascites before and six months after peritoneovenous shunting. # Mean ± S.D. § According to Pugh's classification, see Ref (11).

wedged and free hepatic venous pressures (hepatic venous pressure gradient) was measured with a 7F catheter (cordis Sa, Miami, FL) introduced into a right hepatic vein under fluoroscopy; the wedged position of the catheter was checked as previously described with contrast medium[13]. The zero reference level was arbitrarily positioned at the midaxillary line level. Hepatic blood flow was estimated by the indocyanine green continuous infusion method[14]. Hepatic blood flow was calculated only in patients in whom hepatic indocyanine green extraction was more than 10%. Cardiac output was measured by the thermodilation method with a Swan-Ganz catheter; this catheter was used for measurements of right atrial pressure, mean pulmonary artery pressure and pulmonary wedged pressure. Azygos blood flow was measured with a continuous thermodilution catheter (Webster Laboratories, Altadena, CA) introduced into the arch of the azygos vein; the details have been previously described[15]. During this procedure, arterial pressure was measured by an external monitoring sphygmomanometer (Dinamap, Critikon Inc. Tampa, FL), and heart rate was determined by continuous ECG monitoring. Mean arterial pressure (MAP) was electronically integrated. Systemic vascular resistance (SVR) was calculated according to the following formula:

$$\text{SVR} = (\text{MAP} - \text{right atrial pressure}) \times 80/\text{cardiac output}.$$

No complication occurred in any patients during the hemodynamic investigation.

### *Statistical Analysis*

The results are expressed as mean ± SD. The Student's t test for paired data was used for statistical comparisons.

### RESULTS

### *Comparison of the Hemodynamic Values Between Responsive and Refractory Ascites (Table 2)*

Hepatic venous pressure gradient was lower in patients with refractory ascites than in those with responsive ascites. This resulted from a high free hepatic venous pressure in patients with refractory ascites. Cardiac output in the refractory ascitic patients was significantly lower than in the responsive group. Hepatic and azygos blood flows were not significantly different between the two groups.

### *Comparison of the Hemodynamic Values before and 6 Months after Peritoneovenous Shunting in Patients with Refractory Ascites (Table 2)*

Surgical peritoneovenous shunting was effective in the management of ascites in all patients and patency of the shunt was assessed by shuntography as previously described[16]. Shuntography was performed by transcutanoous puncture of the venous catheter with a 21-gauge needle. Ten milliliters of contrast medium were slowly injected, and the progression was supervised on a television screen. In a normally functioning shunt, contrast medium was washed out in less than 15 seconds. Six months after peritoneovenous shunting, all patients were in good

**Table 2** Splanchnic and systemic hemodynamic in cirrhotic patients with ascites and six months after peritoneovenous shunting

| | | | Refractory ascites | | |
|---|---|---|---|---|---|
| | *Responsive ascites* | *p* * | *Initial evaluation* | *Six months after peritoneovenous shunting* | *p* † |
| Number of patients | 15 | | 14 | 14 | |
| Wedged hepatic venous pressure (mmHg) | 32.0 ± 5.1 | NS | 31.3 ± 6.8‡ | 25.4 ± 5.9 | <0.05 |
| Free hepatic venous pressure (mmHg) | 8.8 ± 4.4 | <0.05 | 12.5 ± 6.3 | 7.6 ± 3.7 | <0.05 |
| Hepatic venous pressure gradient (mmHg) | 23.3 ± 4.1 | < 0.02 | 18.5 ± 5.8 | 17.8 ± 6.1 | NS |
| Hepatic blood flow (L/min) | 0.78 ± 0.18 § | NS | 1.11 ± 0.49 ‖ | 1.50 ± 0.69 | NS |
| Azygos glood flow (L/min) | 0.61 ± 0.29 ¶ | NS | 0.72 ± 0.32 ** | 0.34 ± 0.17 | <0.05 |
| Heart rate (beats/min) | 93 ± 10 | NS | 81 ± 9 | 77 ± 9 | NS |
| Cardiac output (L/min) | 7.05 ± 2.26 | <0.05 | 5.81 ± 1.40 | 6.55 ± 1.75 | <0.05 |
| Arterial pressure (mmHg) | 95.5 ± 12.16 | NS | 91.9 ± 13.8 | 96.2 ± 12.6 | NS |
| Systemic vascular resistance (dyn.s.cm$^{-5}$) | 1069 ± 222 | NS | 1116 ± 494 | 1498 ± 688 | NS |
| Pulmonary arterial pressue (mmHg) | 14.3 ± 4.1 | NS | 15.9 ± 6.9 | 15.3 ± 5.9 | NS |
| Capillary pulmonary pressure (mmHg) | 6.6 ± 3.4 | NS | 8.7 ± 5.7 | 7.6 ± 2.7 | NS |

* Statistical analysis between responsive and refractory ascites. † Statistical analysis between refractory ascites before and six months after peritoneovenous shunting. ‡ Mean ± S.D. § Measured in 5 patients. ‖ Measured in 11 patients. ¶ Measured in 12 patients. ** Measured in 6 patients.

condition without detectable ascites at ultrasonograpahic examination. None had diuretic requirement. Hemodynamic investigations demonstrated a significant decrease in both wedged and free hepatic venous pressure. Hepatic venous pressure gradient was not different from preoperative values. Azygos blood flow significantly decreased by 45% after peritoneovenous shunting. Hepatic blood flow increased by 35%; this was not however significant. Cardiac output significantly increased in all patients after shunting.

### DISCUSSION

The present study suggest that hemodynamic alterations in patients with refractory ascites result from an increase in abdominal pressure and that peritoneovenous shunting improves both splanchnic and systemic hemodynamics by relieving abdominal pressure and enhancing venous return.

It has been postulated that, in patients with cirrhosis, the hyperkinetic syndrome was related to the degree of portosystemic shunting and to the severity of liver disease[5,6]. Although refractory ascites is usually considered a feature of severe liver disease, patients with refractory ascites in this study did not have a hyperkinetic systemic circulation. Cardiac output and hepatic venous pressure gradient were lower in patients with refractory ascites than in those with responsive ascites. The degree of liver failure and abdominal pressure could account for these differences. Liver failure was more severe in patients with responsive ascites than in those with refractory ascites. On the other hand, in patients with refractory ascites, the ascites was more prolonged and more compressive as assessed by the high free hepatic venous pressure than in the patients with responsive ascites. It had been postuled that ascites may also have a negative influence on cardiac function in patients with cirrhosis[17,18]. This is based on clinical and experimental observations. In patients with cirrhosis, cardiac output increased during paracentesis together with a decrease in inferior vena cava and free hepatic venous pressures[17–20]. A decrease in intraabdominal pressure has been proposed as the cause of these hemodynamic alterations[21].

This change, however, was transient. Moreover, it has been demonstrated in the dog that increasing abdominal pressure, by infusing fluid into the abdomen, produced an increase in inferior vena cava pressure and a decrease in cardiac output[20,22,23]. These previous observations support the concept that chronic tense refractory ascites increases intraabdominal pressure and decreases cardiac output and venous return. Thickness of the retroperitoneal space observed in patients with prolonged ascites, might further increase the pressure on the inferior vena cava. In addition, intravascular fluid depletion induced by repeated diuretic treatment may contribute to the decrease in cardiac output[24].

Short-term effects of peritoneovenous shunting were an increase in plasma volume and a dramatic increase in cardiopulmonary pressures and cardiac output[8,9]. It probably accounted for the beneficial effect on peripheral flow and improvement in renal function[8]. It has been demonstrated that these hemodynamic changes were of short duration and that hemodynamic values returned to preoperative measurements within 2 weeks of the operation[8]. Long-term effects of peritoneovenous shunting on systemic and portal hemodynamics have not been well documented. Greig *et al.*[10] observed no significant increase in cardiac output in a small series of patients studied at different postoperative intervals (1 to 15 months). They, however, found a persistent decrease in wedged hepatic venous pressure[8,10]. In the present study, all patients have been investigated on the sixth postoperative month, and no death or shunt obstruction occurred during this period. The two major long-term consequences of shunting were a marked increase in cardiac output and a decrease in wedged hepatic venous pressure. These results are consistent with a reduction of abdominal pressure and an increase in cardiac venous return. Following peritoneovenous shunting, the reduction of abdominal pressure is prolonged, and permanent reinfusion of the ascitic fluid prevents hypovolemia. In contrast, immediately after paracentesis, the increase in cardiac output is transient. The return to initial values could be due to either a reaccumulation of ascites, hypovolemia, or both[21].

It has recently been postulated that peritoneovenous shunting may produce a transient increase in portal pressure and then favour esophageal variceal bleeding[9,25–28]. The long-term measurements in this series of patients showed, in contrast, that wedged hepatic venous pressure significantly decreased after peritoneovenous shunt. This might be associated with a decrease in hepatic vascular resistance since total hepatic blood flow increased by 35%. Although the latter value was not significant because of the wide range of variations, it could indicate a better liver perfusion. The enhancement of liver perfusion could explain the improvement of liver biochemical tests recently observed in patients after peritoneovenous shunt[29]. Lastly azygos blood flow, also a reflection of superior portacaval collateral circulation, significantly decreased 6 months after peritoneovenous shunting. This reduction is in agreement with both the decrease in wedged hepatic venous pressure and the increase in hepatic blood flow. The decrease in wedged hepatic venous pressure and in azygos blood flow observed 6 months after peritoneovenous shunting, support the hypothesis that the risk of esophageal variceal bleeding is not augmented.

*Acknowledgments*

The authors thank Drs. R. Cerini, D. Grange, A. Koshy, R. Moreau and Y. Ozier for their collaboration and Mrs. C. Bertin and C. Girod, Mr. A. Vrolant for their technical collaboration.

Dr A. Hadengue held a fellowship from the Foundation pour la Recherche Médicale and Dr S.S. Lee held a fellowship from the Medical Research Council of Canada.

*References*

1. Murray, J.F., Dawson, A.M. and Sherlock, S. (1958) Circulatory changes in chronic liver disease. *Am. J. Med.*, **24**, 358–67
2. Bayley, T.J., Segel, N. and Bishop, J.M. (1964) The circulatory changes in patients with cirrhosis of the liver at rest and during exercise. *Clin. Sci.*, **26**, 227–35
3. Kontos, H.A., Shapiro, W., Mauck, H.P. and Patterson, J.L. (1964) General and regional circulatory alterations in cirrhosis of the liver. *Am. J. Med.*, **37**, 526–35
4. Valla, D., Poynard, T., Bercoff, E., Bataille, C., Goldfarb, G. and Lebrec, D. (1984) Le syndrome d'hypercinésie circulatoire systémique chez les malades atteints de cirrhose. Relations avec l'insuffisance hépatocellulaire et l'hypertension portale. *Gastroentérol Clin. Biol.*, **8**, 321–4
5. Braillon, A., Lee, S., Girod, C., Peignoux-Martinot, M., Valla, D. and Lebrec, D. (1986) Role of portasystemic shunts in the hyperkinetic circulation of the portal hypertensive rat. *J. Lab. Clin. Med.*, **108**, 543–8

6. Braillon, A., Cales, P., Valla, D., Gaudy, D., Geoffroy, P. and Lebrec, D. (1986) Influence of the degree of liver failure on systemic and splanchnic heamodynamics and on response to propranolol in patients with cirrhosis. *Gut*, **27**, 1204–9
7. Lebrec, D., Kotelanski, B. and Cohn, J.N. (1979) Splanchnic hemodynamic factors in cirrhosis with refractory ascites. *J. Lab. Clin. Med.*, **93**, 301–9
8. Blendis, L.M., Greig, P.D., Langer, B., Baigrie, R.S., Ruse, J. and Taylor, B.R. (1979) The renal and hemodynamic effects of the peritoneovenous shunt for intractable hepatic ascites. *Gastroenterology*, **77**, 250–7
9. Samanta, A.K. and Leevy, C.M. (1989) Effect of peritoneo-venous shunt on portal pressure. *Gut*, **30**, 86–9
10. Greig, P.D. Blendis, L.M. Blendis, B., Taylor, B.R. and Colapinto, R.F. (1981) Renal and hemodynamic effects of the peritoneoveous shunt. II. Long-term effects. *Gastroenterology*, **80**, 119–25
11. Pugh, R.N.H., Murray-Lyon, I.M., Dawson, J.L. and Williams, R. (1973) Transection of the oesophagus for bleeding oesophageal varices. *Br. J. Surg.*, **60**, 646–9
12. Smadja, C. and Franco, D. (1985) The LeVeen shunt in the elective treatment of intractable ascites in cirrhosis: a prospective study of 140 patients. *Ann. Surg.*, **201**, 488–96
13. Valla, D., Bercoff, E., Menu, Y., Bataille, C. and Lebrec, D. (1984) Discrepancy between wedged hepatic venous pressure and portal venous pressure after acute propanolol administration in patients with alcoholic cirrhosis. *Gastroenterology*, **86**, 1400–3
14. Caesar, J. Shaldon, S., Chiandusi, L., Guevara, L. and Sherlock, S. (1961) The use of indocyanine green in the measurement of hepatic blood flow and as a test of hepatic function. *Clin. Sci.*, **21**, 43–57
15. Cales, P.L., Braillon, A., Jiron, M.I., Lebrec, D. (1984) Superior portosystemic collateral circulation estimated by azygos blood flow in patients with cirrhosis. Lack of correlation with esophageal varices and gastrointestinal bleeding. Effect of propranolol. *J. Hepatol.*, **1**, 37–46
16. Smadja, C., Tridard, D. and Franco, D. (1986) Recurrent ascites due to central venous thrombosis after peritoneojugular (LeVeen) shunt. *Surgery*, **100**, 535–41
17. Guazzi, M., Polese, A., Magrini, F., Fiorentini, C., and Olivari, M.T. (1975) Negative influences of ascites on the cardiac function of cirrhotic patients: *Am. J. Med.*, **59**, 165–70
18. Knauer, C.M. and Lowe, H.M. (1967) Hemodynamics in the cirrhotic patient during paracentesis. *N. Engl. J. Med.*, **276**, 191–6
19. El-Raddawi, A., Blumenkehl, M. and Levendoglu, H. (1987) Favorable effects of large volume paracentesis on cardiac and renal functions in patients with tense ascites refractory to conventional treatment. *Gastroenterology*, **92**, 1381 (abst)
20. Henriksen, J.H., Stage, J.G., Schlichting, P. and Winkler, K. (1980) Intraperitoneal pressure: ascitic fluid and splanchnic vascular pressures, and their role in prevention and formation of ascites. *Scand. J. Clin. Lab. Invest.*, **40**, 493–502
21. Cade, R., Wagemaker, H., Vogel, S., Mars, D., Hood-Lewis, D., Privette, M., Peterson, J., Schlein, E., Hawkins, R., Raulerson, D. and Campbell, K. (1987) Hepatorenal syndrome. Studies of the effect of vascular volume and intraperitoneal pressure on renal and hepatic function. *Am. J. Med.*, **82**, 427–38
22. Simon, D.M., McCain, J.R., Bonkovsky, H.L., Wells, J.O., Hartle, D.K. and Galambos, J.T. (1987) Effects of therapeutic paracentesis on systemic and hepatic hemodynamics and on renal and hormonala function. *Hepatology*, **7**, 423–9
23. Barnes, G.E., Laine, G.A., Giam, P.Y., Smith, E.E., Granger, H.J. (1985) Cardiovascular responses to elevation of intraabdominal hydrostatic pressure. *Am. J. Physiol.*, **248**, R208–R213
24. Cereda, J.M., Roulot, D., Braillon, A., Moreau, R., Koshy, A. and Lebrec, D. (1989) Reduction of portal pressure by acute administration of furosemide in patients with alcoholic cirrhosis. *J. Hepatol*, **9**, 246–51
25. Greenlee, H.R., Stanley, M.M. and Reinhardt, G.F. (1981) Intractable ascites treated with peritoneovenous shunts (Le Veen). A 24- to 64-month follow-up of results in 52 alcoholic cirrhotics. *Arch. Surg.*, **116**, 518–23
26. Bernhoft, R.A., Pellegrini, C.A. and Way, L.W. (1982) Peritoneovenous shunt for refractory ascites. Operative complications and long-term results. *Arch. Surg.*, **117**, 631–4
27. Fulenwider, J.T., Galambos, J.D., Smith, R.B., Henderson, J.M. and Warren, W.D. (1986) Le Veen vs denver peritoneovenous shunts for intractable ascites of cirrhosis. *Arch. Surg.*, **121**, 351–5
28. Bories, P., Garcia Compean, D., Michel, H., *et al.* (1986) The treatment of refractory ascites by the Le Veen shunt. A multi-centre controlled trial (57 patients). *J. Hepatol.*, **3**, 212–8

29. Franco, D., Meakins, J.L., Wu, A., Smadja, C., Bonnet, P., Gouffier, E. and Campillo, B. (1989) Long-term results (> 5 years) in patients with peritoneovenous shunting for intractable ascites: liver function and cancer mortality. *HPB Surgery*, **1**, 185–194

*(Accepted by S. Bengmark 15 July 1990)*

### INVITED COMMENTARY

The clinical problems of tense ascites (pain, respiratory distress, immobility, cosmetic deformity, etc.) have been known for centuries. In 1975, Guazzi and co-workers[1] drew attention to the potential harmful effect of ascitic fluid on systemic hemodynamics. The short term effects of peritoneovenous (PV) shunting on hemodynamics and neuro-humoral regulatory systems (increased cardiac output, decreased portal pressure, suppression of the renin angiotensin aldosterone system and the sympathetic nervous system, increased plasma atrial natriuretic factor) are now well established[2–5]. In contrast, we know less of the long term effects[6]. This is in part due to the fact that most patients with decompensated cirrhosis and refractory ascites are poor survivors, and a number of complications, including shunt occlusion, are common after insertion of a PV shunt[5]. In the present study by Vons and co-workers all patients survived and the shunts remained open for at least six months. This provides an outstanding opportunity for long-term study of pathophysiology. The main conclusion is that six months after PV shunting splanchnic- and systemic hemodynamics became similar to those values observed in other patients without ascites. Azygos blood flow, wedged and free hepatic venous pressures decreased, and cardiac output increased significantly. It is important to notice that the azygos venous blood flow decreased in spite of an unchanged hepatic venous pressure gradient. This may be due to a greater influence of the absolute pressure level of the splanchnic venous bed (wedged hepatic vein pressure) than of the hepatic venous pressure gradient. This may simply indicate that the absolute pressure is close to the hydrostatic pressure drop from the esophageal varices to the right atrium. The presence of ascitic fluid will due to its hydrostatic effect increase that pressure[7]. Consequently, amelioration of ascites and thereby decrease of the wedged hepatic vein pressure may reduce the risk of esophageal bleeding independently of any change in the hepatic venous pressure gradient[8].

It is important to stress that the principles of PV shunting are 1° *abdominal decompression* in addition to 2° *central blood volume expansion*[2–5,9]. Previous studies applying plasma volume expansion alone[10] or abdominal decompression alone[11] have not provided good results. The mechanisms by which the combined effect of abdominal decompression and blood volume expansion exert their effects are not fully understood. In this context, it should be recalled that decompression of the renal veins may improve kidney function[12,13]. It should also be recognized that in addition to a long term effect of a well-functioning PV shunt, spontaneous improvement of cirrhotic patients including reduction of wedged hepatic vein pressure may take place[14]. Finally, decreased systemic vascular resistance plays a centrol role in genesis and perpetuation of hepatic ascites[15]. In the present study arterial blood pressure and systemic vascular resistance increased somewhat although not significantly. PV shunting might be combined with vasopressor drugs in order to normalize systemic hemodynamics and thereby renal perfusion pressure[16].

The present investigation shows that several patients with refractory ascites and systemic hemodynamic derangement may have a relatively well preserved liver function. This is stressed by the fact that the patients in the refractory group had a lower Pugh score than in the responsive group ($p = 0.1$, Fisher's exact test). A larger number of patients than previously recognised may suffer from "systemic hemodynamic complications" at a stage of their disease where the hepatocellular function is relatively intact.

Controlled trials of patients with refractory ascites, involving randomization either to PV shunting or to intensive diuretic treatment have not documented any superior long term effect of the PV shunt with respect to survival [17,18]. Still the PV shunt offers a rational therapy from a pathophysiological point of view. Technical improvements including reduction of the rate of complications of PV shunting are awaited.

Jens H. Henriksen
Department of Clinical Physiolsogy, 239
Hvidovre University Hospital
DK-2650 Hvidovre, Denmark

*References*

1. Guazzi, M., Polese, A., Magrini, F., Fiorentini, C. and Olivari, M.T. (1975) Negative influences of ascites on the cardiac function of cirrhotic patients. *Am. J. Med.*, **59**, 165–70
2. Blendis, L.M., Greig, P.D., Langer, B., Baigrie, R.S., Ruse, J. and Taylor, B.R. (1979) The renal and hemodynamic effects of the peritoneovenous shunt for intractable hepatic ascites. *Gastroenterology*, **77**, 77: 250–7
3. Blendis, L.M., Sole, D.P., Campbell, P., Lossing, A.G., Greig, P.D., Taylor, B.R. and Langer, B. (1987) The effect of peritoneovenous shunting on catecholamine metabolism in patients with hepatic ascites. *Hepatology*, **7**, 143–8
4. Klepetko, W., Müller, Ch., Hartter, E., Miholics, J., Schwarzt, Ch., Woloszczuk W. and Moeschl, P. (1988) Plasma atrial natriuretic factor in cirrhotic patients with ascites. *Gastroenterology*, **95**, 764–70
5. Epstein, M. (1982) Peritoneovenous shunt in the management of ascites and the hepatorenal syndrome. *Gastroenterology*, **82**, 790–9
6. Greig, P.D., Blendis, L.M., Blendis, B., Taylor, B.R. and Colapinto, R.F. (1981) Renal and hemodynamic effects of the peritoneovenous shunt II. Long-term effects. *Gastroenterology*, **80**, 119–25
7. Henriksen, J.H., Stage, J.G., Schlichting, P. and Winkler, K. (1980) Intraperitoneal pressure: ascitic fluid and splanchnic vascular pressures, and their role in prevention and formation of ascites. *Scand. J. Clin. Lab. Invest.*, **40**, 493–502
8. Iwasuki, S. and Reynolds, T.B. (1973) Effect of increased intra-abdominal pressure on hepatic hemodynamics in patients with chronic liver disease and portal hypertension. *Gastroenterology* **65**, 294–9
9. Samanta, A.K. and Leevy, C.M. (1989) Effect of peritoneo-venous shunt on portal pressure. *Gut*, **30**, 86–9
10. Vlahevic, Z.R., Adham, N.F., Chalmers, T.C., *et al.* (1967) Intravenous therapy of massive ascites in patients with cirrhosis. *Gastroenterology*, **53**, 211–7
11. Ryan, E. and Neale, G. (1980) Tapping ascites. *Br. Med. J.* **281**, 550–1
12. Firth, J.D., Raine, A.E.G. and Ledingham, J.G.G. (1988) Raised venous pressure: a direct cause of renal sodium retention in oedema. *Lancet*, **i**, 1033–6

13. Henriksen, J.H. and Ring-Larsen, H. (1988) Raised renal venous pressure: Direct cause of renal sodium retention in cirrhosis? *Lancet*, i, 112
14. Gluud, C. and Henriksen, J.H., and the Copenhagen Study Group for Liver Diseases (1987) Liver haemodynamics and function in alcoholic cirrhosis. Relation to testosterone treatment and ethanol consumption. *J. Hepatology*, **4**, 168–73
15. Schrier, R.W., Arroyo, V., Bernardi, M., Epstein, M., Henriksen, J.H., Rodés, J. (1988) Peripheral arterial vasodilation hypothesis: a proposal for the initiation of renal sodium and water retention in cirrhosis. *Hepatology*, **8**, 1151–7
16. Lenz, K., Hörtnagl, H., Druml, W., Grimm, G., Laggner, A., Schneeweisz, B. and Kleinberger, G. (1989) Beneficial effect of 8-ornithin vasopressin on renal dysfunction in decompensated cirrhosis. *Gut*, **30**, 90–6
17. Ring-Larsen, H., Siemsen, O., Krintel, J.J., Stadager, C. and Henriksen, J.H. (1989) Denver shunt in the treatment of refractory ascites in cirrhosis. A randomized controlled trial. *Gastroenterology*, **96**, 30
18. Stanley, M.M., Ochi, S., Lee, K.K. *et al.* and the Veterans Administration Cooperative Study on Treatment of Alcoholic Cirrhosis with Ascites. (1989) Peritoneovenous shunting as compared with medical treatment in patients with alcoholic cirrhosis and massive ascites. *J. Engl. J. Med.*, **321**, 1632–8

---

Supported by grants from the Institut National de la Santé et de la Recherche Médicale and Université Paris-XI.

